# Bayesian estimation for the accuracy of three neuropsychological tests in detecting Alzheimer's disease and mild cognitive impairment: a retrospective analysis of the ADNI database

**DOI:** 10.1186/s40001-023-01265-6

**Published:** 2023-10-12

**Authors:** Xiaonan Wang, Fengjie Li, Jiang Tian, Qi Gao, Huiping Zhu

**Affiliations:** 1 Department of Epidemiology and Health Statistics, School of Public Health, Capital Medical University, No. 10, Xi Toutiao You Anmenwai, Beijing, 100069 People’s Republic of China; 2 Beijing Municipal Key Laboratory of Clinical Epidemiology, Capital Medical University, Beijing, People’s Republic of China; 3 Department of Maternal and Child Health, School of Public Health, Capital Medical University, No. 10, Xi Toutiao You Anmenwai, Beijing, 100069 People’s Republic of China; 4 Laboratory for Gene-Environment and Reproductive Health, Laboratory for Clinical Medicine, Capital Medical University, Beijing, People’s Republic of China

**Keywords:** Alzheimer’s disease, Mild cognitive impairment, Bayesian latent class model, Neuropsychological tests, Sensitivity, Specificity

## Abstract

**Background:**

The neuropathological confirmation serves as the gold standard for diagnosing Alzheimer's disease (AD), but it is usually not available to the living individuals. In addition, the gold standard for diagnosing Mild Cognitive Impairment (MCI) remains unclear yet. Neuropsychological testing, such as the Montreal Cognitive Assessment (MoCA), Mini-Mental State Examination (MMSE) and Alzheimer's Disease Assessment Scale-Cognitive Subscale (ADAS-cog), is commonly used tests in identifying AD and MCI, offering convenience, affordability, non-invasiveness, and accessibility in clinical settings. We aimed to accurately evaluate the discriminative ability of the three tests administrated at the same visit simultaneously in detecting AD and MCI due to AD in the absence of a gold standard.

**Methods:**

A total of 1289 participants aged over 65 were included from the baseline visits of Alzheimer’s disease Neuroimaging Initiative. Bayesian latent class models, accounting for conditional dependence between MoCA and MMSE, were conducted to assess the diagnostic accuracy of the three tests for detecting AD and MCI.

**Results:**

In detecting AD, the ADAS-cog had the highest Youden's Index (0.829), followed by the MoCA(0.813) and MMSE(0.796). The ADAS-cog and MoCA showed similar sensitivity (0.922 vs 0.912) and specificity (0.907 vs 0.901), while the MMSE had lower sensitivity (0.874) and higher specificity (0.922). For MCI detection, the ADAS-cog had the highest Youden's Index (0.704) compared to the MoCA (0.614) and MMSE (0.478). The ADAS-cog exhibited the highest sensitivity, closely followed by the MoCA and MMSE (0.869 vs 0.845 vs 0.757), and the ADAS-cog also had good specificity (0.835 vs 0.769 vs 0.721). The estimated true prevalence of AD among individuals aged over 65 was 20.0%, and the estimated true prevalence of MCI due to AD was 24.8%.

**Conclusions:**

The findings suggest that the ADAS-cog and MoCA are reliable tools for detecting AD and MCI, while the MMSE may be less sensitive in detecting these conditions. A large underdiagnosis of the MCI and Alzheimer’s population still remains in clinical screening.

**Supplementary Information:**

The online version contains supplementary material available at 10.1186/s40001-023-01265-6.

## Background

Alzheimer's disease (AD) is a progressive neurodegenerative disorder characterized by a gradual and irreversible decline in cognitive function, accounting for 60% to 80% of dementia [1, 2]. Mild cognitive impairment (MCI) is a clinical stage that falls between normal aging and dementia [3]. Approximately one-third of individuals with MCI progress to AD dementia within 5 years, while some may reverse to normal cognition [1]. AD dementia affects over 55 million individuals worldwide and about 6.5 million Americans aged 65 and older [1, 4]. This leads to increased dependency and disability among the elderly population, contributing significantly to the global burden of non-communicable diseases. The prevalence of AD in individuals aged over 65 is estimated to be around 10.8%, with MCI due to AD ranging from 8 to 12.9% [1, 5]. However, there remained a significant underdiagnosis or misdiagnosis of MCI and AD in clinical screening [1], indicating the need for early accurate detection methods of MCI and AD to ensure timely intervention and appropriate management strategies.

The framework of clinical–biological diagnosis for AD is based on a combination of specific clinical phenotypes and in vivo biomarkers [6]. A purely biological definition is with a low predictive accuracy for AD and insufficient to definitively predict MCI [6], not readily available in clinical screening as well. Neuropsychological testing remains crucial in detecting AD and MCI. It offers convenient, cost-effective and non-invasive screening tools in clinical settings, making it an essential cognitive assessment method compared to biomarkers detection or imaging exams [7–10]. Among these tools, the Mini-Mental State Examination (MMSE), developed by Folstein et al. [11], is a widely used cognitive test and has traditionally been popular in clinical settings. The Montreal Cognitive Assessment (MoCA), created by Nasreddine et al. [12], is gaining recognition as a comprehensive test that covers multiple cognitive domains. Compared to the MMSE, MoCA has shown a greater capacity to detect MCI among patients who complain of cognitive problems [12, 13]. While the MoCA includes additional subtests focusing on attention and executive function, both tests assess focus on similar cognitive abilities related to memory, concentration, language, and orientation to time and place, which are essential for detecting AD and MCI [11, 12]. Additionally, it has been observed that the distributions of MoCA and MMSE scores are highly correlated, particularly in cases of dementia [14]. However, many validation studies have not taken into account the conditional dependence between MoCA and MMSE in the detection of AD and MCI. Another valuable instrument for detecting cognitive dysfunction is the Alzheimer's Disease Assessment Scale-Cognitive Subscale (ADAS-cog) originally developed by Rosen et al. [15]. This tool is known for its reliability and validity in monitoring the progression of cognitive impairment along the continuum of AD dementia [10, 16].

Nevertheless, the gold standard for a definitive diagnosis of AD can only be established through the neuropathological confirmation of amyloid-β plaques and tau neurofibrillary tangles [2]. Unfortunately, obtaining a pathological biopsy for diagnosis is neither practical nor ethical at the patient's life stage. Furthermore, the clinical application of biomarkers in the diagnosis of MCI and AD is not yet fully developed [6]. The accuracy of the neuropsychological tests is typically evaluated by comparing them to imperfect reference standards, of which sensitivity and specificity are inferior to 100% [17–19]. In the absence of a neuropathological gold standard, it is possible to overestimate or underestimate the true sensitivity and specificity of tests [20]. To address this challenge, Dendukuri et al. [21] and Jones et al. [22] introduced the Bayesian latent class model (BLCM). This approach allows the estimation of the true disease prevalence and test properties while accounting for conditional dependence among tests in the absence of a gold reference standard.

To our knowledge, the use of BLCM for evaluating the test accuracy in detecting AD and MCI has not been previously explored in research studies. By considering the conditional dependence between MoCA and MMSE and correcting the imperfect gold standard bias, the results will offer valuable tests for detecting AD and MCI in clinical screening. Therefore, the objective of this study was to evaluate the performance of MoCA, MMSE, and ADAS-cog in distinguishing between (i) AD from MCI and cognitively normal control subjects (CN), and (ii) MCI patients from CN. The findings of this study provide scientific evidence to support for the clinical selection of more accurate neuropsychological tests for the early detection of MCI and AD.

## Materials and methods

### Study design and subjects

The data used in this study were obtained from the Alzheimer’s Disease Neuroimaging Initiative (ADNI) database, which was a collaborative effort funded by the National Institutes of Health, the National Institute on Aging and 20 companies. ADNI aims to investigate the relationships among the clinical, imaging, genetic, and biochemical biomarkers throughout the spectrum of AD, with the goal of improving early detection and tracking of the progression of the disease. Further details about the study design can be found on the official ADNI website (https://adni.loni.usc.edu/). Ethical approval for the ADNI study was obtained from the medical ethics committees of all participating institutions, and written informed consent was obtained from all participants. This retrospective study involving human participants adhered to the ethical standards set forth by the institutional and national research committees and followed the principles outlined in the Helsinki Declaration.

This retrospective analysis utilized the ADNI dataset, which was released on May 5, 2023. The diagnosis of AD in ADNI was based on the National Institute of Neurological and Communicative Diseases and Stroke/Alzheimer's Disease and Related Disorders Association (NINCDS-ADRDA) criteria, and the staging of cognitive impairment was determined using the Clinical Dementia Rating (CDR) scale. All subjects met the general inclusion criteria and exclusion criteria of the ADNI program. The main inclusion criteria for this study were as follows: (a) availability of baseline visit data for each ADNI stage, and (b) participants aged over 65. The only exclusion criterion was the presence of incomplete scoring information on the MoCA, MMSE and ADAS-cog (the 11-item ADAS-cog) for a participant.

The final dataset for analysis included a total of 1,289 participants aged over 65 who had complete data on the MoCA, MMSE, and ADAS-cog score at baseline visits. This dataset comprised 178 individuals with probable AD, 540 with MCI, and 571 CN individuals. In addition, a subsample consisting of 1,111 individuals with MCI and CN was selected to evaluate the accuracy of the three tests in detecting MCI.

### Statistical analysis

The demographic characteristics of the subjects were presented using means and standard deviations (SD) for continuous variables, such as age, education, MoCA score, MMSE score, and ADAS-cog score. Qualitative categorical variables, such as gender, marriage state, ethnicity, and race, were reported as proportions and frequencies.

The BLCMs were employed to evaluate the sensitivity and specificity of the MoCA, MMSE and ADAS-cog in differentiating AD from non-AD (including participants with MCI and CN) and MCI from CN in the absence of a gold standard. The models integrated the observed data and prior knowledge of parameters to estimate the accuracy of three tests and the true prevalence of AD and MCI due to AD. The specific methods are as follows:

### Sample information

The observed samples were from the ADNI dataset. In order to determine the appropriate cut-off points for detecting AD and MCI using the MoCA, MMSE, and ADAS-cog scales, this study employed the consensus criteria in ADNI as a reference standard. Receiver operating characteristic (ROC) curve analyses were conducted to identify the optimal cut-off points. The results revealed that the optimal cut-off points for a positive diagnosis of AD were determined to be 21.5 (with a cut-off value of < 22) for the MoCA, 26.5(< 27) for the MMSE, and 12.8 (≥ 13) for ADAS-cog. Similarly, for MCI, the optimal cut-off points identified as 23.5(< 24) for the MoCA, 28.5(< 29) for the MMSE, and 7.8(≥ 8) for ADAS-cog. These cut-off points were used to classify individuals as either positive or negative for AD or MCI. The frequency of dichotomous results obtained from the three cognitive assessment tests for detecting AD and MCI are present in Tables 1 and 2, respectively, which was the observed data in the BLCMs. More detailed results of the test accuracy based on the observed data in ADNI can be found in the Additional file 1.

**Table 1:** Cross-classified test results of MoCA, MMSE, and ADAS-cog (*n* = 1289)

Test	MMSE = 1^a^		MMSE = 0^b^	
	ADAS-cog = 1^a^	ADAS-cog = 0^b^	ADAS-cog = 1^a^	ADAS-cog = 0^b^
MoCA = 1^a^	185	40	32	82
MoCA = 0^b^	32	72	44	802

According to the pre-specified cut-off values for each test, ^a^ “1” indicates a positive result, indicating the presence of AD, while ^b^ “0” represents a negative result, indicating non-AD individuals, including participants with MCI and CN

**Table 2:** Cross-classified test results of MoCA, MMSE, and ADAS-cog (n = 1111)

Test	MMSE = 1^a^		MMSE = 0^b^	
	ADAS-cog = 1^a^	ADAS-cog = 0^b^	ADAS-cog = 1^a^	ADAS-cog = 0^b^
MoCA = 1^a^	193	52	75	76
MoCA = 0^b^	84	115	112	404

According to the pre-specified cut-off values for each test, ^a^ “1” indicates a positive result, indicating the presence of MCI, while ^b^ “0” represents a negative result, indicating CN

### Prior information

The accuracy parameters in BLCMs, including sensitivity, specificity, prevalence, and co-variances (specifically addressing the conditional dependence between MoCA and MMSE tests), would be estimated by the Bayesian method in detecting AD and MCI. The prior information of the parameters was necessary in Bayesian framework.

We obtained the priors for sensitivity and specificity of each test by previous literatures [23–25], and the priors for prevalence of AD and MCI due to AD were from review reports [1, 5]. The specific details of informative prior distributions for the parameters in the models are presented in Table 3.

**Table 3:** Summary of informative prior distribution inputs in models for detecting AD and MCI

Parameters	Prior distributions	
	For AD detection	For MCI detection
Prevalence	Beta (2.819, 16.021)	Beta (25.422, 281.851)
Sensitivity		
MoCA	Beta (270.154,20.020)	Beta (824.530, 146.329)
MMSE	Beta (464.322,50.770)	Beta (269.497,110.668)
ADAS-cog	Beta (125.247, 13.288)	Beta (35.209, 9.552)
Specificity		
MoCA	Beta (192.121,22.472)	Beta (440.462,117.819)
MMSE	Beta (514.797, 69.080)	Beta (469.554,192.381)
ADAS-cog	Beta (113.235,9.448)	Beta (56.016,11.479)
Covariances^a^	Cd ~ dunif(−1, 1)	
	Cn ~ dunif(−1, 1)	

^*a*^* Cd* and *Cn* refer to the co-variances explaining the conditional dependence between MoCA and MMSE in detecting AD and MCI patients

### Software implementation

The models were simulated for 100,000 iterations to ensure accurate inferences from the Gibbs sampler of the MCMC algorithm. A burn-in adaptation phase of the first 50,000 iterations was used. The posterior distribution was sampled every 10 iterations to reduce the autocorrelation. The means and 95% posterior confidence intervals (CIs) were reported for all parameters [26]. The Youden’s Index for MoCA, MMSE, and ADAS-cog in detecting AD and MCI were calculated using the corresponding mean posterior estimates of sensitivity and specificity, defined as *Youden’s Index* = *Se*_*Mean*_ + *Sp*_*Mean*_*-1*.

Statistical analysis was performed using R (version 4.2.1, R Core Team) for beta distributions, IBM SPSS Statistics for Windows (version 26.0, IBM Crop) for statistical description, and WinBUGS software (version 14.0, BUGS project) for the BLCM modeling.

## Results

### Characteristics of subjects

A total of 1289 participants aged over 65 were included in the analysis. Among them, 571 (44.30%) were classified as CN, 540 (41.89%) as MCI, and 178 (13.81%) as AD based on the NINCDS-ADRDA criteria. In terms of gender distribution, 47.94% were female and 52.06% were male. The age of the subjects ranged from 65 to 91 years, with a mean age of 73.87 ± 5.85 years. The participants’ education years ranged from 8 to 20 years, with a mean education of 16.36 ± 2.53 years. Detailed demographic characteristics of the populations can be found in Table 4.

**Table 4:** The detailed demographic characteristics of the populations diagnosed and classified by the NINCDS-ADRDA criteria in ADNI

Characteristics^ab^	Total sample (*n* = 1289)	Sub-sample (*n* = 1111)	CN (*n* = 571)	MCI (*n* = 540)	AD (*n* = 178)
Age (years, Mean ± SD)	73.87 ± 5.85	73.40 ± 5.71	72.36 ± 5.62	74.51 ± 5.60	76.78 ± 5.92
Gender (N/%)					
Female	618 (47.94)	561 (50.50)	327 (57.27)	223 (41.30)	68 (38.20)
Male	671 (52.06)	550 (49.50)	244 (42.73)	317 (58.70)	110 (61.80)
Education (years, Mean ± SD)	16.36 ± 2.53	16.46 ± 2.50	16.74 ± 2.36	16.18 ± 2.61	15.70 ± 2.63
Ethnical classification (*N*,%)					
Hispanic/Latino	68 (5.28)	60 (5.40)	33 (5.78)	27 (5.00)	8 (4.49)
Not Hispanic/Latino	1216 (94.34)	1047 (94.24)	535 (93.70)	512 (94.81)	169 (94.95)
Unknown	5 (0.38)	4 (0.36)	3 (0.52)	1 (0.19)	1 (0.56)
Racial classification (*N*,%)					
White	1138 (88.29)	977 (87.94)	492 (86.16)	485 (89.82)	161 (90.45)
Black	93 (7.21)	82 (7.38)	51 (8.93)	31 (5.74)	11 (6.18)
Asian	28 (2.17)	23 (2.07)	15 (2.63)	8 (1.48)	5 (2.81)
Others	30 (2.33)	29 (2.61)	13 (2.28)	16 (2.96)	1 (0.56)
Marriage state (*N*,%)					
Married	964 (74.79)	809 (72.82)	403( 70.58)	406 (75.19)	155 (87.08)
Widowed	146 (11.33)	129 (11.61)	73 (12.78)	56 (10.37)	17 (9.56)
Divorced	127 (9.85)	124 (11.16)	72 (12.61)	52 (9.63)	3 (1.68)
Never married	45 (3.49)	42 (3.78)	22 (3.85)	20 (3.70)	3 (1.68)
Unknown	7 (0.54)	7 (0.63)	1 (0.18)	6 (1.11)	0
MMSE(Mean ± SD)	27.70 ± 2.56	28.44 ± 1.66	29.03 ± 1.19	27.81 ± 1.85	23.06 ± 2.33
MoCA(Mean ± SD)	23.46 ± 4.20	24.48 ± 3.19	25.88 ± 2.53	23.00 ± 3.14	17.14 ± 4.26
ADAS-cog (Mean ± SD)	9.36 ± 6.39	7.60 ± 4.21	5.60 ± 2.87	9.71 ± 4.38	20.36 ± 6.67

^a^*SD* refers to standard deviation

^b^*N,%* refers to the number of the samples and the corresponding proportion

### Results from Bayesian estimation

**Fig. 1:**
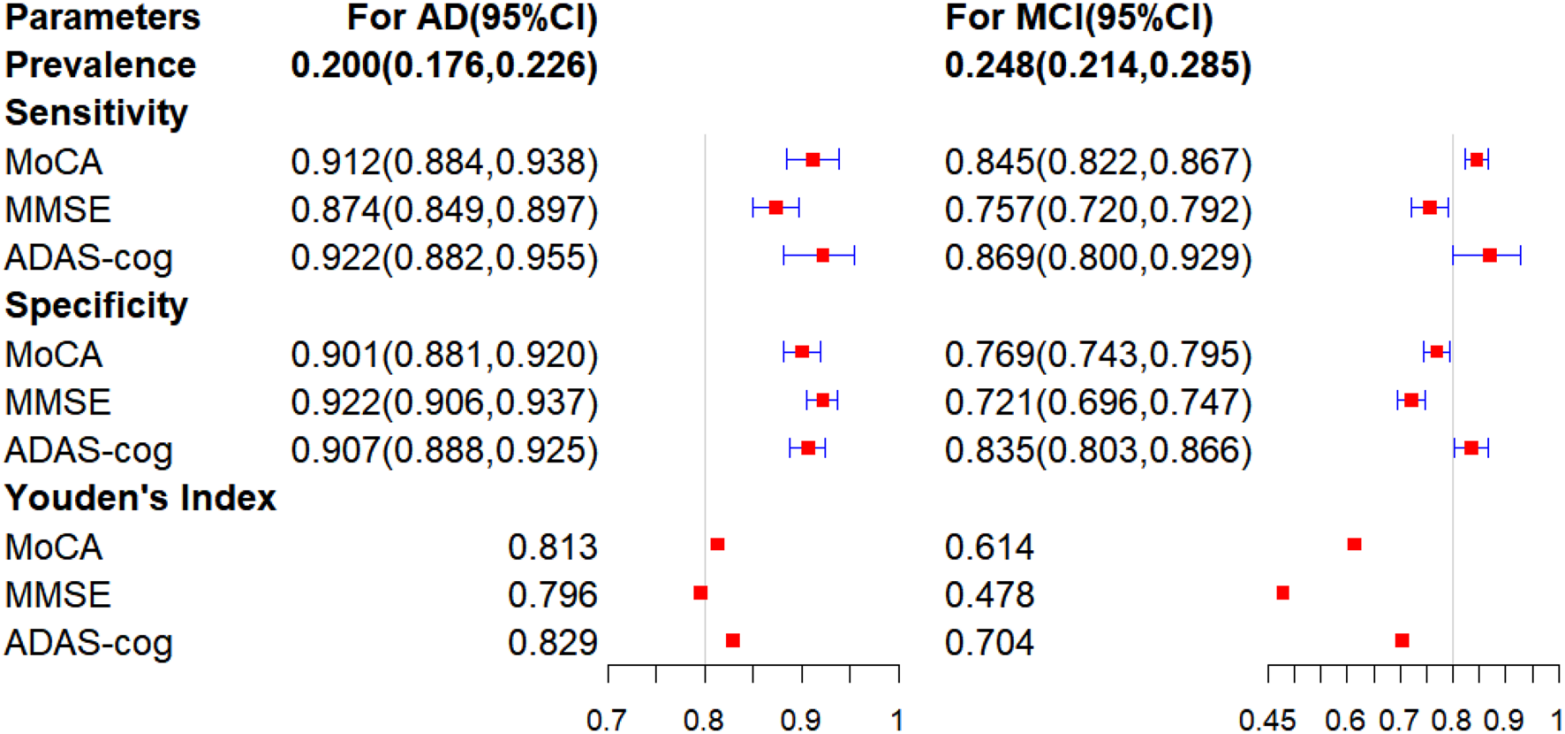
Results of test accuracy for AD and MCI detection from Bayesian estimation. Posterior means and 95% confidence intervals (CIs) for sensitivity and specificity of the MoCA, MMSE, and ADAS-cog in detecting AD and MCI, and the true prevalence of AD and MCI due to AD among the individuals aged over 65, considering the conditional dependencies between MoCA and MMSE

The posterior means and the corresponding 95% CIs for sensitivities and specificities of MoCA, MMSE and ADAS-cog and the true prevalence of AD and MCI due to AD are summarized in Fig. 1.

All three measures have fairly discriminatory abilities in detecting AD, with the ADAS-cog demonstrating the highest Youden's Index of 0.829 followed closely by the MoCA of 0.813 and MMSE of 0.796. Both the sensitivity and the specificity of ADAS-cog and MoCA in detecting AD are relatively similar. Specifically, the ADAS-cog had a sensitivity of 0.922 (95% CI 0.882–0.955) and a specificity of 0.907 (95% CI 0.888–0.925), the MoCA had a sensitivity of 0.912 (95% CI 0.884–0.938) and a specificity of 0.901 (95% CI 0.881–0.920). The MMSE showed a lower sensitivity of 0.874 (95% CI 0.849–0.897) and a better specificity of 0.922 (95% CI 0.906–0.937).

In terms of detecting MCI, the ADAS-cog exhibited a Youden's Index of 0.704, while the MoCA demonstrated a value of 0.614, and the MMSE had a value of 0.478. The ADAS-cog showed the highest sensitivity of 0.869(95% CI 0.800–0.929), closely followed by the MoCA with a sensitivity of 0.845 (95% CI 0.822–0.867). The ADAS-cog also displayed good specificity with a value of 0.835 (95% CI 0.803–0.866), while the MoCA had a specificity of 0.769 (95% CI 0.743–0.795). In contrast, the MMSE demonstrated relatively lower accuracy, with a sensitivity of 0.757 (95% CI 0.720–0.792) and a specificity of 0.721 (95% CI 0.696–0.747).

The estimated true prevalence was 20.0% (95% CI 17.6–22.6%) for AD and 24.8% (95% CI 21.4–28.5%) for MCI due to AD among the population aged over 65.

The autocorrelation checks revealed that the autocorrelation function soon approached zero. The iteration history and the iteration track plots showed stable patterns, indicating convergence of the iterative process. These findings indicate that the MCMC algorithm converged successfully.

## Discussion

In this study, we utilized the BLCMs to assess the discriminative ability of the MoCA, MMSE, and ADAS-cog in distinguishing between (i) patients with AD and individuals with MCI and CN, and (ii) individuals with MCI and CN. This analysis was conducted in the absence of a definitive gold standard for diagnosis. The results revealed several key findings. First, the ADAS-cog demonstrated the highest discriminative ability in detecting both AD and MCI among the three tests, as indicated by the highest Youden’s Index and sensitivity. The MoCA closely followed, showing comparable performance. Second, the MoCA exhibited significantly higher sensitivity than the MMSE in detecting both AD (0.912 vs 0.874) and MCI (0.845 vs 0.757), highlighting its potential as a more sensitive screening tool. Lastly, the prevalence of the Alzheimer's population was found to be higher than previously reported, suggesting a substantial underdiagnosis of AD and MCI due to AD in clinical screening.

Overall, previous studies have consistently supported the superiority of the MOCA over the MMSE in various comparisons, including differentiating AD from MCI patients, AD from CN individuals, AD and MCI from CN individuals, as well as differentiating MCI from CN individuals [27–32]. Our findings align with these studies as we observed that the MoCA exhibited higher sensitivity and Youden’s index compared to the MMSE when distinguishing between AD and non-AD individuals, as well as between MCI from CN individuals in the absence of a gold standard. However, the MMSE demonstrated slightly higher specificity in detecting AD. In addition, the results of this study in the ADNI population are consistent with the findings of previous meta-analyses that investigated the diagnostic performance of the MOCA and MMSE for detecting AD and MCI due to AD under non-gold standard conditions using a hierarchical Bayesian latent class meta-analysis [23, 24]. In that study, the MoCA demonstrated a sensitivity of 0.934 and a specificity of 0.899 in detecting AD, while the MMSE had a sensitivity of 0.883 and a specificity of 0.903 [23]. For the detection of MCI, the MoCA showed a sensitivity of 0.85 and a specificity of 0.79, whereas the MMSE had a sensitivity of 0.71 and a specificity of 0.71 [24].

It is noteworthy that the ADAS-cog exhibited the highest Youden's Index in detecting both AD and MCI patients, with values of 0.829 and 0.704, respectively. These values were significantly higher compared to the MoCA and MMSE. Few studies have directly compared the accuracy of ADAS-cog with the MMSE and MoCA in tracking cognitive decline in AD or MCI. However, existing evidence suggests that the ADAS-cog is useful in differentiating MCI patients from normal elderly individuals [33] and has high predictive validity in both MCI and AD [9, 25]. For example, Jemaa et al. [34] reported that the Arabic version of the ADAS-cog had excellent discriminant power, with 83% sensitivity and 85% specificity at a cut-off of 11 for diagnosing AD. The ADAS-Cog demonstrated superior performance compared to the MMSE in detecting both AD (AUC: 0.927 vs 0.899) and MCI patients (AUC: 0.835 vs 0.769) [9]. Notably, the MoCA performed equally well as the ADAS-Cog in detecting AD [9]. It was worth mentioning that the sensitivity and the specificity of MoCA [27, 29], MMSE [35] or ADAS-cog [9, 10, 25] were superior in detecting AD than detecting MCI in different populations or language versions, such as in Spanish [27, 29], Dutch [35], Portuguese [9], Chinese [10]. This conclusion aligns with the findings of our study, where a gold standard for AD and MCI was absent in ADNI participants from the North American population.

In this study, we determined the optimal cut-off values using ROC curve analysis and applied the values to generate the binary results of each test in identifying AD and MCI. The test performance and the cut-offs for positivity of the MoCA, MMSE and ADAS-cog for different populations were influenced by educational level, age, and gender [36–38]. The ADAS-cog utilized cut-off points of > 9 for MCI and > 12 for AD, while the MMSE used cut-off points of < 29 for MCI and < 26 for AD [9]. Our study also confirmed the psychometric validity and the reliability of the ADAS-Cog as an instrument for assessing cognitive function in AD and MCI patients, with cut-off values of ≥ 8 for MCI and ≥ 13 for AD. The optimal cut-off value of ADAS-cog for AD was set at 13 generating 89% specificity and 90% sensitivity [39]. In the previous study, an education-adjusted MoCA score of 24 and of 22 was optimal for maximizing test performance when detecting MCI and AD [36], which was equal to the optimal cut-off values from the ROC curve analysis. The MoCA continued to exhibit superior performance compared to the MMSE in screening for MCI when different cut-off values were applied based on age groups (57–64, 65–69, 70–76, 77–80, and 81–97 years old) and educational levels (0–6, 7–9, and over 10 years), or among the individuals aged 55 to 80 with less than 5 years of education [40, 41]. Besides, in the three age groups (60–79, 80–89, and ≥ 90 years old), the optimal MoCA cut-off scores for detecting MCI were ≤ 25,  ≤ 24, and ≤ 23, respectively, and for detecting dementia were ≤ 24,  ≤ 21, and ≤ 19, respectively, which demonstrated relatively higher diagnostic accuracy for detecting MCI and dementia compared to the MMSE[38].

Totally, the ADAS-cog told more information and more sensitive about the true disease status of AD and MCI, and the MoCA always outperformed the MMSE both for identifying AD or MCI patients in clinical, while the MMSE was more useful to rule out the non-AD. Our results are consistent with most studies, even at different ages, different levels of education, and different cut-offs of tests for different populations.

According to the literature reviews, about 10.8% of people aged over 65 has AD, and the prevalence rate of MCI due to AD was 8–12.9% [1, 5]. While the estimated true prevalence in this study was 20.0% for AD and 24.8% for MCI due to AD. The results suggested that a large underdiagnosis of the MCI and Alzheimer’s population still remained in clinical screening as reported in America [1]. Therefore, clinical screening by accurate method especially for MCI should be strengthened to ensure early detection of cognitive impairment patients.

To the best of our knowledge, this is the first original research on application of BLCM to simultaneously evaluate the accuracy of MoCA, MMSE, and ADAS-cog for AD and MCI test diagnostics. Given the difficulty of establishing the true disease status of individuals in the absence of a perfect reference test for the progression of AD, BLCM can be used to justify the imperfect reference standard bias. Dendukuri and Joseph [21] also indicated that adjusting for the possibility of conditional dependence among diagnostic tests had a significant impact on the posterior estimates of the prevalence, sensitivity, specificity, and covariances directly. It is also the first validation study to consider the conditional dependence between MoCA and MMSE for the detection of AD and MCI in the absence of a gold standard. The BLCM combined prior information with the observed data through Bayesian modeling to conduct a posterior inference of parameters [21, 42]. In this study, the posterior confidence interval of test accuracy was relatively concentrated than in previous analyses.

However, there are several limitations in this study. First, we compared the accuracy of the three tests to detect AD and MCI in the absence of a gold standard using the Bayesian model in this study, but did not consider the influence of covariates on test scores. Future studies should aim to construct a Bayesian model that incorporates covariates to estimate the true disease prevalence, as well as the sensitivity and specificity of neuropsychological tests. This would provide a more comprehensive understanding of the diagnostic accuracy in the presence of covariates without a gold standard. Second, biomarkers are currently accepted as an effective clinical diagnosis method for the definition of AD and the pre-dementia stages [6]. However, we did not focus on the diagnostic accuracy of biomarkers. Neuropsychological tests still play an important role in detecting AD and MCI, so we mainly aimed to compare the sensitivity and specificity of MoCA, MMSE, and ADAS-cog for the detection of AD and MCI in the absence of a gold standard. Additionally, obtaining biomarker data for large populations during clinical screening can be challenging. Previously, study conducted by Barrado et al. [43] had an estimation of diagnostic accuracy of a combination of continuous biomarkers in ADNI allowing for conditional dependence between the biomarkers and the imperfect reference test in detecting AD. Future research could consider using the BLCM method to estimate the diagnostic accuracy of a combination of the biomarkers and the neuropsychological tests for AD and MCI. Third, to assess the consistency of the results obtained using the BLCM method as a surrogate for the pathological gold standard, a verification study with autopsy confirmed patients should be conducted. However, the validation sample in ADNI was relatively small. Out of the 69 patients following the guidelines for neuropathological assessment of AD by the National Institute on Aging-Alzheimer's Association, only five were diagnosed as non-AD, while the remaining 64 patients had Alzheimer disease neuropathologic changes (ADNC). Further research with a larger and more diverse sample is needed to validate these findings and strengthen the reliability of the conclusions.

## Conclusions

In conclusion, the BLCMs reveal that the ADAS-cog and the MoCA are reliable tools for detecting AD and MCI. The ADAS-cog is the most informative with the highest Youden’s index and sensitivity for the detection of AD and MCI. The MoCA exhibits significantly higher sensitivity than the MMSE in detecting both AD and MCI. A large underdiagnosis of the MCI and Alzheimer’s population still remains in clinical screening.

### Supplementary Information

**Additional file 1: Figure S1.** Results of the Accuracy of Three Neuropsychological Tests in Detecting AD and MCI from Observed Data in ADNI.

## Data Availability

The datasets used and analyzed during the current study are available from the corresponding author on reasonable request.

## Abbreviations

AD: Alzheimer's disease
MCI: Mild cognitive impairment
MMSE: Mini-Mental State Examination
MoCA: Montreal cognitive assessment
ADAS-cog: Alzheimer's disease assessment scale-cognitive subscale
BLCM: Bayesian latent class model
CN: Cognitively normal controls
ADNI: Alzheimer’s disease neuroimaging initiative
NINCDS-ADRDA: National Institute of Neurological and Communicative Disorders and Stroke and the Alzheimer’s Disease and Related Disorders Association
CIs: Confidence intervals
AUC: Area under the curve

## References

[1] Alzheimer’s Association (2023). Alzheimer's disease facts and figures. Alzheimers Dement.

[2] Lane CA, Hardy J, Schott JM (2018). Alzheimer's disease. Eur J Neurol.

[3] Petersen RC, Doody R, Kurz A (2001). Current concepts in mild cognitive impairment. Arch Neurol.

[4] Alzheimer's Disease International, World Alzheimer Report 2022: Life after diagnosis: Navigating treatment, care and support. 2022. https://www.alzint.org/u/World-Alzheimer-Report-2022/.

[5] Yuan L, Chaojie L, Dehua Y (2021). Prevalence of mild cognitive impairment in community-dwelling Chinese populations aged over 55 years: a meta-analysis and systematic review. BMC Geriatr.

[6] Dubois B, Villain N, Frisoni GB (2021). Clinical diagnosis of Alzheimer's disease: recommendations of the International Working Group. Lancet Neurol.

[7] Matías-Guiu JA, Valles-Salgado M, Rognoni T (2017). Comparative diagnostic accuracy of the ACE-III, MIS, MMSE, MoCA, and RUDAS for screening of Alzheimer disease. Dement Geriatr Cogn Disord.

[8] Hemmy LS, Linskens EJ, Silverman PC (2020). Brief Cognitive tests for distinguishing clinical Alzheimer-type dementia from mild cognitive impairment or normal cognition in older adults with suspected cognitive impairment: a systematic review. Ann Intern Med.

[9] Joana N, Sandra F, Diana D, Jorge A, Isabel S (2018). Validation study of the Alzheimer's disease assessment scale-cognitive subscale (ADAS-Cog) for the Portuguese patients with mild cognitive impairment and Alzheimer's disease. Clin Neuropsychol.

[10] Yang H, Cheng Z, Li Z (2019). Validation study of the Alzheimer's disease assessment scale-cognitive subscale for people with mild cognitive impairment and Alzheimer's disease in Chinese communities. Int J Geriatr Psychiatry.

[11] Folstein MF, Folstein SE, McHugh PR (1975). “Mini-mental state”. A practical method for grading the cognitive state of patients for the clinician. J Psychiatr Res.

[12] Nasreddine ZS, Phillips NA, Bédirian V (2005). The Montreal cognitive assessment, MoCA: a brief screening tool for mild cognitive impairment. J Am Geriatr Soc.

[13] Ganguli M, Blacker D, Blazer DG (2011). Classification of neurocognitive disorders in DSM-5: a work in progress. Am J Geriatr Psychiatry.

[14] Trzepacz PT, Hochstetler H, Wang S, Walker B, Saykin AJ (2015). Relationship between the Montreal cognitive assessment and mini-mental state examination for assessment of mild cognitive impairment in older adults. BMC Geriatr.

[15] Rosen WG, Mohs RC, Davis KL (1984). A new rating scale for Alzheimer's disease. Am J Psychiatry.

[16] Hani ZN, Eveline S, Lh LL, Nagaendran K (2016). Psychometric properties of Alzheimer's disease assessment scale-cognitive subscale for mild cognitive impairment and mild Alzheimer's disease patients in an Asian context. Ann Acad Med Singapore.

[17] Gaugler JE, Kane RL, Johnston JA, Sarsour K (2013). Sensitivity and specificity of diagnostic accuracy in Alzheimer's disease: a synthesis of existing evidence. Am J Alzheimers Dis Other Demen.

[18] Prestia A, Caroli A, Wade SK (2015). Prediction of AD dementia by biomarkers following the NIA-AA and IWG diagnostic criteria in MCI patients from three European memory clinics. Alzheimers Dement.

[19] Rockwood K (2010). Con: Can biomarkers be gold standards in Alzheimer's disease?. Alzheimers Res Ther.

[20] Umemneku Chikere CM, Wilson K, Graziadio S, Vale L, Allen AJ (2019). Diagnostic test evaluation methodology: a systematic review of methods employed to evaluate diagnostic tests in the absence of gold standard—an update. PLoS ONE.

[21] Dendukuri N (2001). Bayesian approaches to modeling the conditional dependence between multiple diagnostic tests. Biometrics.

[22] Jones G, Johnson WO, Hanson TE, Christensen R (2010). Identifiability of models for multiple diagnostic testing in the absence of a gold standard. Biometrics.

[23] Wang X, Li F, Gao Q (2022). Evaluation of the accuracy of cognitive screening tests in detecting dementia associated with Alzheimer's disease: a hierarchical Bayesian latent class meta-analysis. J Alzheimers Dis.

[24] Wang X, Li F, Zhu H, Jiang Z, Niu G, Gao Q (2022). A Hierarchical Bayesian latent class model for the diagnostic performance of mini-mental state examination and Montreal cognitive assessment in screening mild cognitive impairment due to Alzheimer's disease. J Prev Alzheimers Dis.

[25] Park SH, Han K (2022). Is the Alzheimer's disease assessment scale-cognitive subscale useful in screening for mild cognitive impairment and Alzheimer's disease? A systematic review. Curr Alzheimer Res.

[26] Enøe C, Georgiadis MP, Johnson WO (2000). Estimation of sensitivity and specificity of diagnostic tests and disease prevalence when the true disease state is unknown. Prev Vet Med.

[27] Delgado C, Araneda A, Behrens MI (2019). Validation of the Spanish-language version of the Montreal cognitive assessment test in adults older than 60 years. Neurologia (Barcelona, Spain).

[28] Li X, Jia S, Zhou Z (2018). The role of the Montreal Cognitive Assessment (MoCA) and its memory tasks for detecting mild cognitive impairment. Neurol Sci.

[29] Aguilar-Navarro SG, Mimenza-Alvarado AJ, Palacios-García AA (2018). Validity and reliability of the spanish version of the Montreal Cognitive Assessment (MoCA) for the detection of cognitive impairment in Mexico. Revista colombiana de psiquiatria.

[30] Ciesielska N, Sokołowski R, Mazur E (2016). Is the Montreal Cognitive Assessment (MoCA) test better suited than the Mini-Mental State Examination (MMSE) in mild cognitive impairment (MCI) detection among people aged over 60?. Meta-analysis. Psychiatr Pol.

[31] Roalf DR, Moberg PJ, Xie SX (2013). Comparative accuracies of two common screening instruments for classification of Alzheimer's disease, mild cognitive impairment, and healthy aging. Alzheimers Dement.

[32] Pinto TCC, Machado L, Costa MLG (2019). Accuracy and psychometric properties of the Brazilian version of the Montreal Cognitive Assessment as a brief screening tool for mild cognitive impairment and Alzheimer's disease in the initial stages in the elderly. Dement Geriatr Cogn Disord.

[33] Pyo G, Elble RJ, Ala T, Markwell SJ (2006). The characteristics of patients with uncertain mild cognitive impairment on the Alzheimer disease assessment scale-cognitive subscale. Alzheimer Dis Assoc Disord.

[34] Jemaa SB, Romdhane NA, Bahri-Mrabet A (2017). An Arabic version of the cognitive subscale of the Alzheimer’s disease assessment scale (ADAS-Cog): reliability, validity, and normative data. J Alzheimer's Dis.

[35] Sleutjes DKL, Harmsen IJ, van Bergen FS (2020). Validity of the Mini-Mental state examination-2 in diagnosing mild cognitive impairment and dementia in patients visiting an outpatient clinic in the Netherlands. Alzheimer Dis Assoc Disord.

[36] Pugh EA, Kemp EC, van Dyck CH, Mecca AP, Sharp ES (2018). Effects of normative adjustments to the Montreal cognitive assessment. Am J Geriatr Psychiatry.

[37] Pin NT, Lei F, Shiong LW (2015). Montreal Cognitive Assessment for screening mild cognitive impairment: variations in test performance and scores by education in Singapore. Dement Geriatr Cogn Disord.

[38] Tan J-P, Li N, Gao J (2015). Optimal cutoff scores for dementia and mild cognitive impairment of the Montreal cognitive assessment among elderly and oldest-Old Chinese population. J Alzheimers Dis.

[39] Lakshminarayanan M, Vaitheswaran S, Srinivasan N (2022). Cultural adaptation of Alzheimer's disease assessment scale-cognitive subscale for use in India and validation of the Tamil version for South Indian population. Aging Ment Health.

[40] Parunyou J, Sookjaroen T, Solaphat H (2015). The Montreal cognitive assessment-basic: a screening tool for mild cognitive impairment in illiterate and low-educated elderly adults. J Am Geriatr Soc.

[41] Mellor D, Lewis M, McCabe M (2016). Determining appropriate screening tools and cut-points for cognitive impairment in an elderly Chinese sample. Psychol Assess.

[42] Johnson WO, Jones G, Gardner IA (2019). Gold standards are out and Bayes is in: implementing the cure for imperfect reference tests in diagnostic accuracy studies. Prev Vet Med.

[43] Garcia Barrado L, Coart E, Burzykowski T (2017). Estimation of diagnostic accuracy of a combination of continuous biomarkers allowing for conditional dependence between the biomarkers and the imperfect reference-test. Biometrics.

