# Supplementary material for: Bayesian estimation for the accuracy of three neuropsychological tests in detecting Alzheimer's disease and mild cognitive impairment: a retrospective analysis of the ADNI database
Source: Eur J Med Res. 2023 Oct 12;28:427. doi: 10.1186/s40001-023-01265-6 (PMC10568914; doi:10.1186/s40001-023-01265-6)
Supplement: Supplementary file 1 — Additional file 1: Figure S1. Results of the Accuracy of Three Neuropsychological Tests in Detecting AD and MCI from Observed Data in ADNI. [file 40001_2023_1265_MOESM1_ESM.docx]

***Bayesian Estimation for the Accuracy of Three*** ***Neuropsychological Tests in Detecting Alzheimer's Disease and Mild Cognitive Impairment: A Retrospective Analysis of the ADNI Database***

Results from Observed Data in ADNI

The ADAS-cog MoCA, MMSE, and tests demonstrated good discrimination between the AD and non-AD groups (MCI and CN) when using the the consensus criteria based on the NINCDS-ADRDA criteria as the reference standard. The area under the curve (AUC) values were 0.921, 0.961, and 0.955 for the MoCA, MMSE, and ADAS-cog, respectively (Fig S1.A). In terms of detecting MCI, the ADAS-cog had the highest AUC value of 0.790, followed by the MoCA with an AUC of 0.762, and the MMSE with an AUC of 0.706 (Fig S1.B).


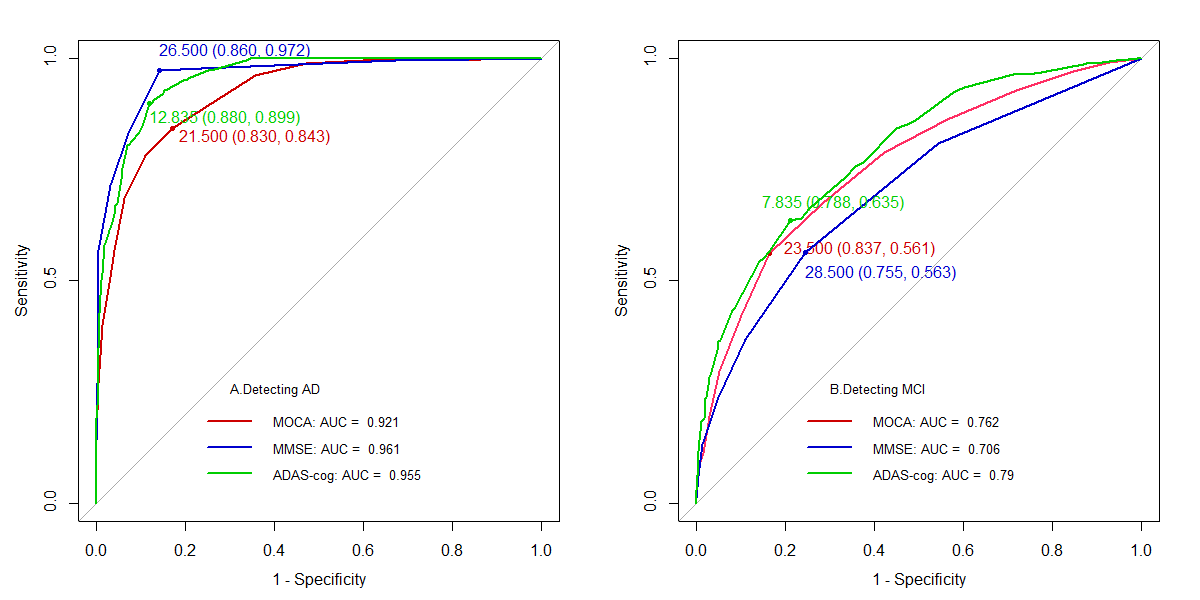


Fig S1 ROC curves of the MoCA, MMSE and ADAS-cog in detecting AD (A) and MCI (B), taking the consensus criteria based on the NINCDS-ADRDA criteria as the reference standard. The points on the ROC curves represent the optimal cut-off values (sensitivity, specificity).
